# Bringing Patient-Centered Care to the Fore in Diseases of the Pancreas

**DOI:** 10.1155/2015/459214

**Published:** 2015-05-14

**Authors:** Sayali A. Pendharkar, Maxim S. Petrov

**Affiliations:** Department of Surgery, University of Auckland, Auckland 1142, New Zealand

## Abstract

Diseases of the pancreas are often very challenging for both patients and doctors as well as pose a considerable burden on healthcare system. Emerging evidence on the importance of shared-decision making in medicine stresses the need to integrate best clinical evidence and patient-reported outcomes to deliver optimal patient care. This paper argues that patient-centered care should no longer be a hermit in management of pancreatic diseases in the 21st century.

## 1. Introduction

Pancreatic diseases constitute most commonly of acute pancreatitis, chronic pancreatitis, and pancreatic cancer and affect more than 330,000 people each year in the United States alone [1]. The economic burden of acute pancreatitis, the most frequent disease of the pancreas, is estimated in the United States to be $2.6 billion per year for inpatient costs, with the overall hospital mortality rate at 1.0% [1]. Chronic pancreatitis, although far less common than acute pancreatitis, has much worse long-term outcomes with a 50% mortality rate within 20 to 30 years from diagnosis and an increased risk of developing pancreatic cancer [2]. With mortality rate almost equal to the incidence rate due to the rapid fatality in most cases, pancreatic cancer remains one of the worst prognosis malignancies. After colorectal cancer, pancreatic cancer is the next most common gastrointestinal cancer in the United States, affecting over 40,000 people annually [1].

**Figure 1:**
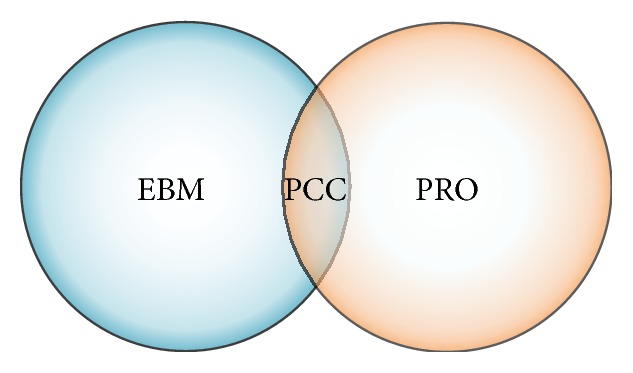
Conceptual framework of patient-centered care. EBM: evidence-based medicine; PCC: patient-centered care; PRO: patient-reported outcomes.

In recent years, patient-centered care has emerged as an important new paradigm in clinical management in general and care of patients with pancreatic diseases in particular [3, 4]. Nonetheless, patients' perspectives, values, and preferences are poorly incorporated in clinical decision making. Recent literature highlights the need to integrate evidence-based medicine and patient-reported outcomes in clinical care of patients [3, 5] and the conceptual framework is schematically presented in Figure 1.

## 2. Fledging Concept of Patient-Centred Care

Recent advances in pancreatic diseases, though often resulting in improved clinical outcomes, have inadvertently created a health care environment in which patients are excluded from important discussions and decision making processes. Complete information transfer from the physician to the patient, about how their problems are being managed and how to navigate through the many diagnostic and treatment options available, is often lacking [6]. Although patient well-being has always been the ultimate objective of medicine, the historical perspective has been to provide a paternalistic, provider-centred model of care. Hippocrates endorsed a way of practice that encouraged “concealing most things from the patient while attending to him… revealing nothing of the patient's future or present condition” [7]. In 1871, a Boston physician and poet Oliver Holmes also advised that “patient has no more right to all the truth you know than he has to all the medicine in your saddlebags… he should only get so much as is good for him,” essentially reiterating the Hippocratic approach to patient care [8]. However, over the ensuing decades, medicine has become increasingly patient-centred with the emphasis shifting from patient compliance to patient participation.

The concept of medicine being patient-centred has been a constituent of the patients' rights movement since the 1960s [8]. Patient-centred medicine, as a term, was coined in 1969 by Enid Balint, a British psychoanalyst. However, as a concept, it was brought to light after numerous landmark legal cases had emphasised a patient-centred standard. The* Canterbury v. Spence* case established the need for informed consent and patient's right to it [9]. In late 1970s, widely publicised cases such as* Quinlan *and* Cruzan* triggered considerable interest in advance directives, following which physicians began to share information with patients and include them and their families in the decision [10]. In the United States, these cases led to the development of Patient Self-determination Act introduced and enacted in 1989 and 1991, respectively. This legislation mandated that healthcare institutes advise patients on their right to accept or decline medical care [10].

The paradigm of patient-centered care or “narrative medicine” [11] is acquiring increased prominence along with personalized medicine and tailored therapeutics interventions to better manage diseases of the pancreas [12, 13]. The Institute of Medicine defined patient-centered care as “care that is respectful of and responsive to individual patient preferences, needs and values” ensuring that “patient values guide all clinical decisions” [6]. This definition largely coincided with the then modern mores of patient-centered care described by Slack and Kassirer in 1977 and 1983, respectively. While the former urged physicians to recognize patients' rights to make their own medical decisions, the latter advocated that physicians, setting aside their supreme authority in making life-and-death decisions, should instead undertake “the less glamorous and more time-consuming process of exploring optimal outcomes with the patient” [14, 15].

Nowadays, patient-centered care focuses on assessing and improving patients' daily health outcomes by taking into account the patients' own objectives, values, and preferences. These include physical functionality, symptoms, quality of life (QoL), social status, and emotional status, which all fall within “goal-oriented patient care outcomes” [4]. To date, quality of care assessments and health outcomes have not incorporated patient-centeredness. While quality of care has addressed disease-specific care and preventive processes, outcomes measure has concentrated on short- and long-term condition-specific indicators in addition to overall mortality. Unfortunately, these measures and processes are only good for relatively healthy patients with no comorbidities and do not address patients with severe disability, multiple conditions, and/or short life expectancy, which are especially common in chronic pancreatitis and pancreatic cancer [16]. The overall quality of care does not solely depend on disease-specific care processes and, hence, may not accurately reflect the effects of treatments on patients with comorbidities [17].

An alternative approach to improved care would be to determine a patient's “individual health goals within or across a variety of dimensions” [17]. This approach is potentially advantageous because of the following reasons:

1. The discussion focuses on individual needs and desires instead of on universal health states.
2. Goal-oriented patient care allows patients with comorbidities to focus on outcomes spanning across several conditions and accordingly align treatments towards common goal(s).
3. This approach helps patients articulate health states important to them and their priority such as a choice between receiving hospice care or aggressive treatment [17].

The goal-oriented patient care thus allows physicians and patients to collaborate on those health states most desired by the patients, agree on treatment measures most suitable to achieve these goals, and monitor their progress.

The concept of patient-centered care, though diametrically different from the established model of “illness-oriented care,” iterates the importance of focusing medical attention on individual patient's requirements as opposed to the doctor's [18]. However, not all patient goals may be attainable or realistic. In this case, the physician needs to explain the plausible measures and negotiate with the patient potentially achievable goals [17]. This highlights the need for “shared-decision making” whereby the patient and the clinician coexist in a social, therapeutic, and economic relation of “mutual and highly interwoven prerogatives” [18]. Implementation of patient-centered care will further result in cost-effective management of pancreatic diseases, thereby reducing the burden on healthcare systems. Focusing on the outcomes most desirable to patients may require fewer resources than traditional disease-specific care.

## 3. Measuring Patient-Reported Outcomes

Moving towards patient-centered care requires the use of quality instruments that adequately capture patient goal elicitation and attainment [17]. The gradual shift towards patient-centered care needs to be vigorously paralleled with application and study of “softer” outcomes such as a patient's functional status, health-related QoL, and satisfaction.

With the growing importance of patient-centered care, it is imperative to consider patients' QoL in management of pancreatic diseases. Since the 1980s, it has been increasingly realised that the traditional, biologically based end-points such as morbidity and mortality alone do not adequately represent the potential outcomes of medical interventions. Health status measurement has evolved and broadened the term QoL, to encompass patient experiences regarding functionality, mood, life satisfaction, cognition, and ability to fulfil social, family, and occupational roles [19]. To quote C.A. O'Boyle: “The QoL construct may be viewed as a paradigm shift since it shifts the focus of attention from symptoms to functioning and establishes the primacy, or at least the legitimacy, of the patient perspective” [19].

The QoL measures aim to describe patient-perceived health and social status of a population, compare interventions, determine the cost and benefits of medical treatments and health policies, and thereby improve the delivery of care [20]. Despite the literature presenting enough evidence in support of the benefits associated with routine assessment of patients' QoL in clinical practice and research, sufficient empirical evidence is lacking in pancreatic diseases [21]. The QoL questionnaires constitute the commonly administered instrument to measure QoL. QoL is comprised of multiple factors including but not limited to physical limitations, functionality, spiritual beliefs, satisfaction, and psychological well-being. The following subsections detail some of the most commonly used QoL questionnaires.

### 3.1. The Short-Form 36 Health Survey (SF-36)

The SF-36 questionnaire, developed in the United States, was first administered in the Rand Corporation's Health Insurance Experiment [22]. Accessible in 120 languages and utilised globally to gauge the health of diverse populations [23–26], it is a measure of patient-perceived health status [22] and has increasingly been classified as the research-validated gold standard [26]. The questionnaire constitutes 35 questions with one transition question assessing patient-perceived change in general health over the past year. The remaining questions are divided into eight categories addressing limitations in physical function, role physical, bodily pain, general health, vitality (fatigue and energy), social functioning, and role emotional and mental health. Scores obtained from these subscales are then summarised into the Physical or Mental Component Summary scores (PCS or MCS, resp.) [22]. The scores are assigned on a scale of 0–100 (0: worst health; 100: best health) and the overall score reflects patient-reported perception of the overall health status [22]. 

SF-12 is a succinct version of the original SF-36 questionnaire. It refers solely to the PCS and MCS scores as determinants of QoL. The summary scores are also assigned on a scale of 0–100 with higher scores representing better QoL. Despite being a concise questionnaire, it helps distinguish between patients with chronic medical conditions and those without [27].

### 3.2. The European Organisation for Research and Treatment of Cancer Quality of Life Questionnaire (EORTC-QLQ)

Conventionally a cancer-specific QoL tool designed for self-administration [28], EORTC-QLQ was developed by the EORTC QoL Study Group. It is a reliable and cross-culturally valid instrument (validated across more than 24 countries) which comprises five function-specific and three symptom-specific scales [29]. 

The EORTC-QLQ-C30 questionnaire is a revised and improved version of the first-generation EORTC-QLQ-C36 questionnaire developed in 1987 [28]. Lung cancer patients, from Western Europe, Australia, North America, and Japan, were recruited in the initial phase of instrument development and validation given the high incidence and rapid progression of the disease [28]. The C30 questionnaire consists of multi-item and single item scales thereby reflecting the multidimensionality of QoL. The five functional scales asses a patient's physical functionality, role physical, emotional health, social functioning, and cognitive ability while the three symptom-specific scales assess fatigue, pain, and nausea and vomiting. The questionnaire also incorporates a global health and QoL scale. Additional symptoms reported commonly by cancer patients, such as loss of appetite, diarrhoea, constipation, dyspnoea, and sleep disturbance, and perceived financial impact, are assessed by the remaining single item scales [28]. Scores are recorded on a scale of 0–100 with a high score indicating a higher QoL. The scores are calculated as per the EORTC-QLQ-C30 scoring manual [29]. The EORTC-QLQ-C30 has been translated into eight languages and supplements specific to various cancer types added to make the questionnaire more relevant. These second- and third-generation questionnaires have been widely administered in randomised controlled trials (RCT). For example, the EORTC-QLQ-BR23, developed and validated for breast cancer [30], was used in an RCT studying effects of resistance exercise on QoL and fatigue in breast cancer patients [31]. Results from the questionnaire were beneficial and showed that QoL was maintained and fatigue mitigated in these patients during chemotherapy [31]. The EORTC-QLQ-STO22, for gastric cancer, was developed and validated as part of a study from 2001 to 2003 [32]. This supplement accounted for gastric cancer-specific symptoms and issues, namely, abdominal dysphagia, reflux, and eating restrictions, as well as chemotherapy and radiation-related symptoms [32]. 

One such supplement was developed for patients suffering from pancreatic cancer. The EORTC-QLQ-PAN26 has been used extensively in pancreatic cancer trials. A few examples are the phase III trial comparing gemcitabine to a PEFG infusion [33] or the phase II trial studying gemcitabine versus capecitabine in patients with advanced cholangiocarcinoma and carcinoma of the gallbladder [34]. The PAN26 supplement includes 26 items addressing disease and treatment-related symptoms and associated emotional consequences. The disease and treatment-related symptom scales assess pain, digestive symptoms, flatulence, indigestion, hepatic symptoms, cachexia, altered bowel habits, and side effects. Body image, concern of future health, sexuality, and healthcare satisfaction are assessed by emotional consequences-related scales [33].

### 3.3. The Sickness Impact Profile (SIP)

The SIP instrument was developed by Bergner and colleagues in 1981 [35] and is a measure of behavioural changes in patients consequential to a disease. As standardized questionnaire, it constitutes 136 statements categorised into 12 subcategories which assess the dysfunction experienced in day-to-day activities due to a given illness. The physical domain addresses the following three categories: ambulation, body care and movement, and mobility, whereas the psychosocial dimension addresses emotional behaviour, social interaction, communication, and alertness behaviour [35]. SIP further assesses sleep and rest, work, home management, eating habits, recreation, and pastimes. The scores obtained are expressed as a percentage of likely disability (0%: no health-related impairment; 100%: total incapacity). Numerically, a score of 0–10 indicates patients doing well in their life; a score of 10–20 illustrates mild illness-related dysfunctions; and a score of >20 demonstrates evident disability in daily activities [35].

### 3.4. The Karnofsky, Eastern Cooperative Oncology Group (ECOG) Score, and Rankin Scores

The Karnofsky and Rankin Scores instrument was first developed in 1949 by Karnofsky. While the tool does measure physical ability, it fails to measure a patient's psychosocial condition. For the physical domain, the score is ranged from 0 to 5 (0: no symptoms and normal activity; 5: severe physical disability and need for continuous nursing support with bed-rest) [36]. The overall score is then converted to a scale of 0–100 (0: death; 100: ability to conduct normal daily physical activities) [37].

The ECOG score is a widely used performance status scale for evaluating the functional status of a cancer patient [38]. Developed initially in 1960, the score is reported on a scale of 0–4 (0: fully active and able to carry on all predisease performance without restriction; 4: unable to get out of bed) with lower score indicating better performance [39]. The ECOG scale is an important factor in determination of prognosis in numerous malignancies such as breast cancer, small cell lung cancer, ovarian cancer [40], and pancreatic cancer [41]. The ECOG scale is reliable and valid and can also be used to determine a patient's eligibility for recruitment into clinical trials [40].

### 3.5. The Function Assessment of Chronic Illness Therapy (FACIT) Scale Tool 

FACIT scale tool differs from the above-mentioned QoL instruments in that it is a collection of several health-related QoL questionnaires with the aim of managing chronic illnesses. The tool was developed in 1987 and includes 27 questions compiled into the following four categories: physical well-being, emotional well-being, social/family well-being, and functional well-being. The FACIT instrument presents over 40 different scales and all measures have undergone a standard scale development and validation methodology [42]. All FACIT scales have been developed such that a high score reflects better QoL in patient [43].

### 3.6. The Rosser Disability and Distress Index

The Rosser Disability and Distress Index instrument is based on degree of distress and disability as experienced by the patient. It combines the distress and disability scores in order to provide an accurate level of disease severity. The numeric scale assigns a score of either 0 or 1 (0: severe illness or death; 1: perfect health) [44]. The instrument has been validated in several disease settings [44].

### 3.7. The Hospital Anxiety Depression Scale and the Cantril Ladder

The Hospital Anxiety Depression Scale is administered to assess psychiatric disorders in a population of nonpsychiatric patients who have been hospitalized for somatic reasons. Depression, anxiety, and aggression are the three subscales addressed. The Cantril Ladder, on the other hand, comprises two one-question scales that record points on a scale of 1–10. These determine a patient's life satisfaction at a specific point in time and three years after that. A higher score, as for other QoL tools, indicates better patient outcomes [45].

### 3.8. The Gastrointestinal Quality of Life Index

The Gastrointestinal Quality of Life Index, a reliable and validated bilingual (German and English) instrument developed in 1989, was designed to assess QoL in patients with gastrointestinal diseases [46]. The questionnaire constitutes 36 questions with five possible responses for each question. Each individual response is scored on a scale of 0–4 (0: least desirable; 4: most desirable) with all the responses summed to give an overall numerical score on a scale of 0–144. A higher overall score implies better QoL. However, it is not a diagnostic tool and while it can moderately differentiate between healthy individuals and those with gastrointestinal diseases, it does not distinguish between diseases [47].

### 3.9. The Abdominal Surgery Impact Scale (ASIS)

The ASIS questionnaire was initially designed to assess short-term QoL following abdominal surgery [48]. The ASIS constitutes of 18 questions (scored on a scale ranging from 1 (strongly agree) to 7 (strongly disagree)) categorised into six domains: physical limitations, functional impairment, pain, visceral function, sleep, and psychological well-being. The score can range from 18 to 126. Cronbach's alpha was used to measure the internal reliability of the ASIS instrument in acute pancreatitis. The ASIS was found to be reliable for five out of six domains with the reliability coefficient ranging from 0.761 (pain) to 0.911 (functional impairment). Only the visceral function domain had a lower reliability coefficient at 0.691 [48].

### 3.10. Izbicki Score

The Izbicki pain score is a validated pain score designed specifically for chronic pancreatitis. The score consists of four questions assessing frequency of pain, pain intensity (VAS score), use of analgesics, and disease-related inability to work. The score is reported on a scale of 0–100 (0: no pain; 100: excruciating pain) [49–51].

### 3.11. M-ANNHEIM

M-ANNHEIM is a multiple risk factor classification system developed in 2007 [52]. This system helps categorize patients according to the clinical stage, aetiology, and severity of their disease (chronic pancreatitis). The M-ANNHEIM classification system has taken all previous chronic pancreatitis classifications into consideration before developing a standardized classification system accounting for all possible definitions and symptoms. There is one patient-reported subscale included, patient report of pain, where a patient is asked to choose from one of the following options: no pain without therapy, recurrent acute pancreatitis, no pain with therapy, intermittent pain, and continuous pain [52, 53].

## 4. Conclusion

There is a growing need to integrate patient-reported outcomes and evidence-based medicine to create an environment of shared-decision making and provide optimal patient-centered care, which is essential for quality and economically sustainable health care [5]. Utilising health-related QoL questionnaires to assess patient-reported outcomes and determine patients' preferences will allow clinicians and patients to embark on a joint venture to shared-decision making. Development and implementation of clinical practice guidelines in the field of pancreatic diseases that formulate recommendations based on both best clinical evidence and patient-reported outcomes could be a step forward in bringing patient-centered care to the fore.
